# Data for zonisamide effect on length and weight of 14 day-old rat pups and abortion rate and day in refractory epileptic pregnant rats

**DOI:** 10.1016/j.dib.2018.01.010

**Published:** 2018-01-20

**Authors:** Reza Narenji Sani, Keivan Keramati, Niloufar Saberi, Melika Moezifar, Ali Mahdavi

**Affiliations:** a Department of Clinical Sciences, Faculty of Veterinary Medicine, Semnan University, Semnan, Iran; b Department of Basic Sciences, Faculty of Veterinary Medicine, Semnan University, Semnan, Iran; c Department of Basic Sciences, Faculty of Veterinary Medicine, University of Tehran, Tehran, Iran; d Department of Animal Sciences, Faculty of Veterinary Medicine, Semnan University, Semnan, Iran

## Abstract

Length and weight of 14 day-old rat pups and also abortion rate and day on refractory epileptic pregnant rats after treatment with zonisamide (ZNS) are presented. Lamotrigine-resistant chemical kindling procedure was used for inducing of refractory epilepsy. For further interpretation follow the research article: Effect of zonisamide on refractory epilepsy during pregnancy in lamotrigine resistant kindled rats (Sani et al., 2017) [1].

**Specifications Table**

**Table:** 

Subject area	Neuropharmacology
More specific subject area	Refractory epilepsy
Type of data	Figure and table
How data was acquired	Digital caliper, digital scales, Progesterone ELISA Kit (IBL international GMBH, Germany)
Data format	Analyzed
Experimental factors	Refractory epilepsy was induced in each rats before pregnancy and after that zonisamide treatment was performed
Experimental features	LTG-resistant chemical kindling procedure was used for inducing of refractory epilepsy [2]. Length and weight of rat pups was measured and abortion rate and day was examined by serum progesterone concentration measurement
Data source location	Shahmirzad, Semnan, Iran
Data accessibility	The data are available with this article

**Value of the data**

• The data can be used to understand zonisamide effect on abortion rate and day in refractory epileptic pregnant rats.
• The data can be used to understand zonisamide effect on rat pups growth during pregnancy in refractory epileptic pregnant rats.

## 1 Data

**Fig. 1:**
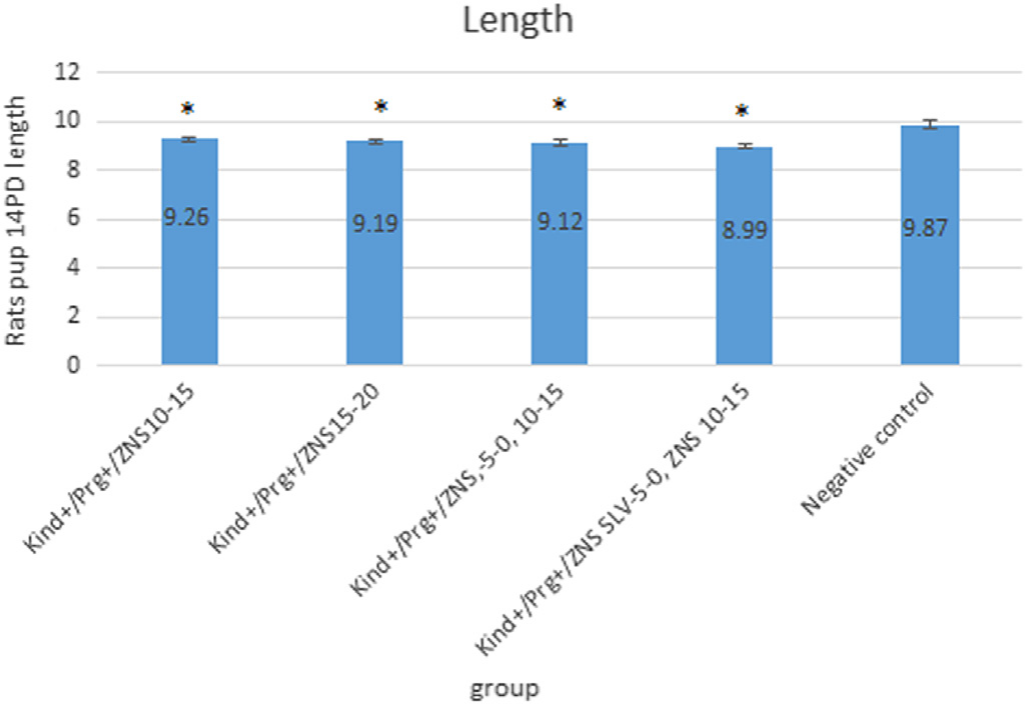
Length of rat pups in 14PD in pregnant treatment groups and negative control group in Wistar rats. ZNS (30 mg/kg) was used on LTG-resistant kindled (Kind+) pregnant (Prg+) rats in day 10–15 (in rats with or without ZNS or methanol and ethyl acetate as a ZNS SLV challenge between days − 5 to 0) or 15–20 of pregnancy and ZNS SLV was used in day 10–15 of pregnancy. Also, negative control group included intact pregnant rats without any kindling stimulation and treatment. The value is expressed as the mean ± SEM from all the pregnant groups (n = 8) except for the Kind+/Prg+/ZNS SLV 10–15 group. *: Compared to negative control group (α = 0.05). LTG: Lamotrigine, ZNS: Zonisamide, Solvent: SLV, 14PD: 14 Post natal day.

**Fig. 2:**
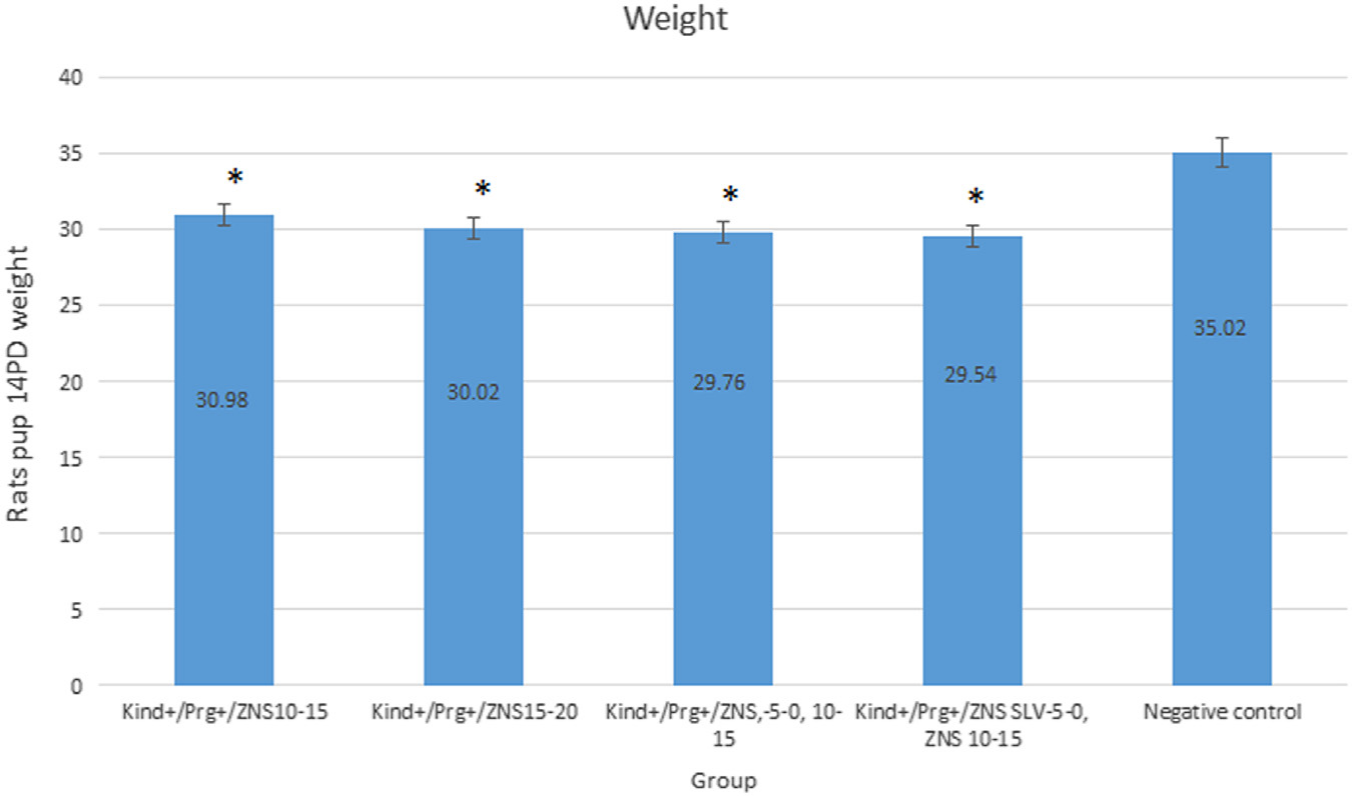
Weight of rat pups in 14PD in pregnant treatment groups and negative control group in Wistar rats. ZNS was used on LTG-resistant kindled (Kind+) pregnant (Prg+) rats in day 10–15 (in rats with or without ZNS or methanol and ethyl acetate as a ZNS SLV challenge between days − 5 to 0) or 15–20 of pregnancy and ZNS SLV was used in day 10–15 of pregnancy. Also, negative control group included intact pregnant rats without any kindling stimulation and treatment. The value is expressed as the mean ± SEM from all the pregnant groups (n = 8) except for the Kind+/Prg+/ZNS SLV 10–15 group. *: Compared to negative control group (α = 0.05). LTG: Lamotrigine, ZNS: Zonisamide, Solvent: SLV, 14PD: 14 Post natal day.

Data provided in this article correspond to rat pups growth during pregnancy in refractory epileptic pregnant rats with zonisamide treatment and also effect of zonisamide on abortion rate and day in refractory epileptic pregnant rats (Fig. 1, Fig. 2) (Table 1).

**Table 1:** Comparison of mean abortion day, abortion rate, Rat pup/Parturition and Rat pup/Mother rat between treatment and negative control groups in Wistar rats.

Group	Mean abortion day	Abortion rate (%)	Rat pup/Parturition	Rat pup/Mother rat
Kind+/Prg+/ZNS10-15	11.66	50 (4/8)^b^	7 (4/28) ^a^	3.5 (28/8) ^b^
Kind+/Prg+/ZNS SLV 10-15, positive control 1	10.33	100 (8/8)^a^	0 (0/0) ^b^	0 (0/8) ^c^
Kind+/Prg+/ZNS15-20	15.66	50 (4/8)^b^	7.5 (4/30) ^a^	3.75 (30/8) ^b^
Kind+/Prg+/ZNS,-5-0, 10-15	11.66	87.5 (6/8)^a^	6.5 (2/13) ^a^	1.5 (13/8)^c^
Kind+/Prg+/ZNS SLV-5-0, ZNS 10-15	11.66	62.5 (5/8)^b^	6.3 (3/19) ^a^	2.37 (19/8) ^b^
Negative control	–	0 (0/8)^c^	6.75 (8/54) ^a^	6.75 (54/8)^a^
SEM	1.66			
P value	0.36	0.04	0.06	0.02

ZNS was used on LTG-resistant kindled (Kind+) pregnant (Prg+) rats in day 10–15 (in rats with or without ZNS or methanol and ethyl acetate as a ZNS SLV challenge between days − 5 to 0) or 15–20 of pregnancy and ZNS SLV was used in day 10–15 of pregnancy. Also, the negative control group included intact pregnant rats without any kindling stimulation and treatment. All of sex pregnant groups were in this table. Eight rats were included in each group. McNemar test was used to analyze abortion rate and analysis of variation and Duncan postcode was used for comparing mean of Rat pup/Parturition and Rat pup/Mother rat parameters. ^abc^ Different letters in a column showed significant differences (α = 0.05).

LTG: Lamotrigine, ZNS: Zonisamide, Solvent: SLV.

## 2 Experimental design, materials and methods

### 2.1 Animals

Wistar rats (200–240 g) were obtained from Shahmirzad Laboratory Animal Research Center in Shahmirzad, Semnan, Iran. Rats were maintained in the animal house under controlled conditions (12 h light-and-dark cycles, at 21 °C with 50% relative humidity) with laboratory chow and water provided ad libitum. Before mating, the experimental, positive and negative control groups were kindled by LTG (Daroupakhsh Co., Iran) resistant model (n = 42). Chemical kindling in rats was induced by giving an i.p. injection of subconvulsant dose of 30 mg/kg Pentylenetetrazol (PTZ) (Sigma, USA), one hour after saline injection, every alternate day for a maximum of 74 days. For induction of LTG-resistant kindled rat, animals received LTG (5 mg/kg i.p.) 1 h before every PTZ challenge every alternate day for a maximum of 74 days and were observed for 30 min; seizure score was recorded according to the modified Racine scale [1,3] as follows: stage 0, no response; stage 1, grooming and hyperactivity; stage 2, head nodding and tremor; stage 3, bilateral forelimb clonus; stage 4, clonus with rearing; and stage 5, clonic seizures with loss of postural control. Rats were considered to be fully kindled when they were observed to have 3 consecutive stage 5 seizures. Drug treatment was discontinued once the rats were fully kindled. Two days following the last kindling session, in both saline and LTG (5 mg/kg i.p.) treated, fully-kindled animals received higher dose of LTG (15 mg/kg i.p) before PTZ challenge and showed stage 5 seizure to verify LTG-resistance. After that, LTG-resistant kindled rats were divided into 7 groups. Drug resistant full kindling day was considered as blocks for dividing.

All of the experimental procedures using animals were approved by the Experimental Animal Ethics Committee of the faculty of veterinary medicine, Semnan University, Semnan, Iran, and performed in accordance with the guidelines for handling laboratory animals.

### 2.2 Study groups

ZNS (Tehrandarou Co., Iran) (30 mg/kg) was used on LTG-resistant kindled pregnant rats in day 10–15 (in rats with or without ZNS or methanol and ethyl acetate as a ZNS (solvent) SLV challenge between days − 5 and 0) or 15–20 of pregnancy and ZNS SLV was used in day 10–15 of pregnancy. Also, negative control group included intact pregnant rats without any kindling stimulation and treatment. Each group included 8 rats.

### 2.3 Length and weight evaluation

Body length, weight of rat pups was evaluated in 14 parturition day (PD). Rat pups were anesthetized via inhalation of ether on 14 PD.

### 2.4 Abortion rate and day

Progesterone is essential to initiate embryo implantation and maintain pregnancy in eutherian mammals. Serum progesterone values between 25 and 35 ng/ml throughout days 10–19 gestation was needed to maintain the pregnancy [4]. Abortion day was assessed by measuring serum progesterone concentration using Progesterone ELISA Kit (IBL international GMBH, Germany).

After analysis of variances (ANOVA), Duncan's multiple range test was used to comparing means as posthoc, the parametric data including rat pup/parturition and rat pup/mother rat ratio, were analysed by ANOVA (in the case of equal repeats) and McNemar tests were used to analyse abortion rate as a non-parametric parameter by using SPSS statistical software (ver: 21, 2012).
