# Supplementary material for: Data for zonisamide effect on length and weight of 14 day-old rat pups and abortion rate and day in refractory epileptic pregnant rats
Source: Data Brief. 2018 Jan 20;17:279–83. doi: 10.1016/j.dib.2018.01.010 (PMC5988214; doi:10.1016/j.dib.2018.01.010)
Supplement: Supplementary file 1 — Transparency document [file mmc1.docx]

There is no conflict of interest
